# Genome Sequence of *Arthrobacter globiformis B-2979* Phage *IsHungry*

**DOI:** 10.17912/micropub.biology.001819

**Published:** 2025-12-22

**Authors:** Ryan Buckner, Karina Chang, Caroline Hannah Chavez, Leon Chen, Owen Chen, Yilin Chen, Noah Cheng, Yihao Fan, Isabelle Chiang, Rena Hwang, Jenny Jung, Alyssa Kim, Cadence Liang, Ryan Moinazad, Rumaysha Momen, Ella Ren, Amy Yao, Christa Bancroft

**Affiliations:** 1 Biological Sciences, University of Southern California, Los Angeles, California, United States; 2 Neuroscience, University of Southern California, Los Angeles, California, United States; 3 Quantitative and Computational Biology, University of Southern California, Los Angeles, California, United States; 4 Gerontology and Quantitative and Computational Biology, University of Southern California, Los Angeles, California, United States; 5 Biological Sciences and Chemistry, University of Southern California, Los Angeles, California, United States; 6 Gerontology, University of Southern California, Los Angeles, California, United States

## Abstract

Phage IsHungry was isolated on *Arthrobacter globiformis B-2979* and has siphovirus morphology. Its genome consists of 40,628 base pairs, encoding 61 putative genes and 3 tRNAs. As is typical of the phages with similar gene content that area assigned to actinobacteriophage FF cluster, all but 4 genes are transcribed unidirectionally.

**Figure 1. Plaque and TEM image of Phage IsHungry:**
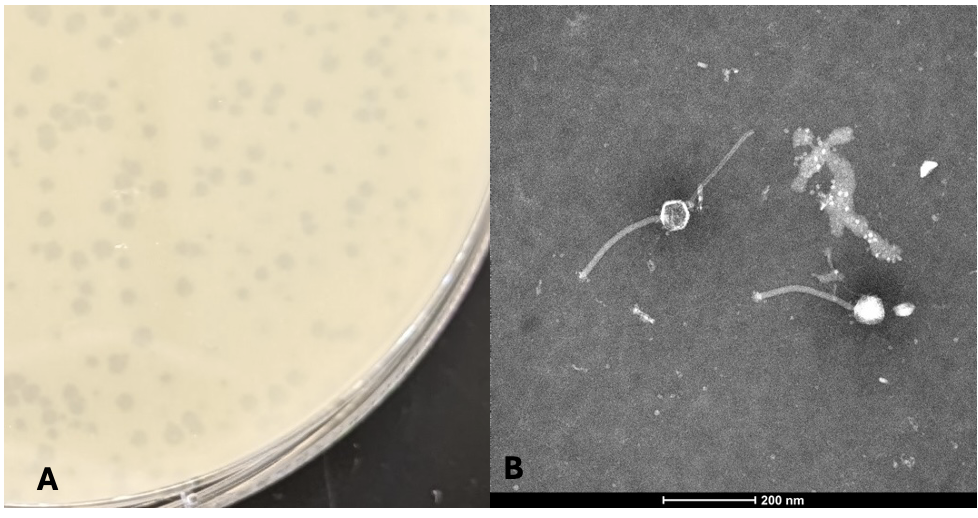
A) Plaques formed by phage IsHungry. B) An electron microscopy (EM) image of the phage particle stained with Nano-W (Nanoprobes).

## Description

As some of the simplest yet most diverse biological entities in the world, bacteriophages have gained relevance today in both clinical and ecological spheres of research (Hatfull 2022). We aimed to discover phages that infect *Arthrobacter* , a common soil bacterium that potentially is useful in bioremediation efforts (Klyczek et al. 2017).

Phage IsHungry was isolated from a sandy soil sample located at the University of Southern California (34.019018 N, 118.290813 W) using standard procedures (Zorawik et al. 2024). &nbsp;Briefly, the soil sample was washed in peptone (15g/L)-yeast extract (1g/L)-calcium (4.5mM) (PYCa) medium with 0.1% dextrose, the wash was filtered (0.22µm), then inoculated with *Arthrobacter globiformis B-2979* and incubated with shaking at 30˚C for 48 h. The culture was refiltered, diluted, and plated on a PYCa agar-covered petri dish with 3mL molten PYCa top agar containing *Arthrobacter globiformis B-2979* (Zorawik et al. 2024). After 24 h at 30˚C, IsHungry produced small, clear, and uniform plaques, with a diameter of 1mm +/- 0.5mm (n=4) &nbsp;(Figure 1a). The phage was purified with three round of picking individual plaques, which were spaced at least 1 cm apart, and plating before a lysate was prepared and used to for negative-stain transmission electron microscopy. IsHungry has siphovirus morphology, possessing a tail 200-203 nm in length and a capsid of 61-63 nm in diameter (n=3) (Figure 1b). Capsid size and tail length were determine by using Adobe Illustrator software (v.26.0.1) and using the ruler tool. &nbsp;We compared the pixel distance length of the legend on the TEM image file to the pixel length of the tail and capsid to determine a relative size.

Genomic DNA was purified from a high titer lysate (4.33 x 10 ^11^ pfu/mL) using the Promega Wizard DNA cleanup kit, prepared for sequencing using the NEB Ultra II FS kit, and sequenced on an Illumina NextSeq 1000 (XLEAP-P1 kit). 100-base single-end raw reads (2,891,419) with 6794 coverage were trimmed with cutadapt 4.7 (using the option: –nextseq-trim 30) and filtered with skewer 0.2.2 (using the options: -q 20 -Q 30 -n -l 50) prior to assembly using Newbler v2.9 and checked for accuracy and genomic termini using Consed v29 (Gordon and Green 2013), as described previously (Russell 2018) yielding a 40,628 bp genome with single-stranded overhangs (5' - TCCGCCGCGTGA - 3') and 65.4% GC-content. The genome sequence was auto-annotated using DNAMaster (v5.23.6 Build 2705) (Pope and Jacobs-Sera 2018) embedded with GeneMark v2.0 (Besemer and Borodovsky 2005) and Glimmer v3.02 (Delcher et al 2007). Following auto-annotation, Starterator Version 1.2 (https://github.com/SEA-PHAGES/starterator) was used to refine start sites. IsHungry encodes 61 putative open reading frames. Three tRNAs (GCT-Arg, TCT-Arg, and CCC-Gly) were identified by Aragorn v1.2.38 (Laslett and Canback 2004) and tRNAscan-SE (Lowe and Eddy 1997). The default parameters were used for all software. Based on gene-content similarity of at least 35% to phages in the Actinobacteriophage database (Russell and Hatfull 2017), IsHungry was assigned to phage cluster FF. HHPred (Söding et al. 2015), NCBI BLAST (Altschul et al. 1990), and Phamerator (database Actino_Draft version 594) (Cresawn et al. 2011) were used to deduce the putative functions of proteins encoded by open reading frames.

As with other cluster FF phages, two tyrosine integrases were identified, suggesting IsHungry may be able to establish lysogeny. This would be consistent with experimentally observed lysogen formation for another cluster FF phage, QuinnAvery (Wise and Sivanathan 2025). These genes are in the center of the genome, one in the forward and one in the reverse direction, flanking a forward arg tRNA. The open reading frames in the left side portion of the FF phage genomes are mostly related to structural components of the phage. The genes in the latter region of cluster FF phages include those associated with DNA metabolism and 16 out of 26 with unknown functions. Across all cluster FF phages, over 88% of the genes are transcribed forward, apart from 4-9 genes in each genome that are transcribed in the opposite direction.

**Nucleotide Sequence Accession and Read Numbers** &nbsp;

IsHungry is available at GenBank accession PV876961 and Sequence Read Archive (SRA) accession SRX28484014 .
